# MitraClip for Severe Mitral Regurgitation from Chordal Rupture during Balloon Aortic Valvuloplasty

**DOI:** 10.14797/mdcvj.1111

**Published:** 2022-09-20

**Authors:** Akiva Rosenzveig, Syed Zaid, Joshua Hsu, Hasan Ahmad, Joshua Goldberg

**Affiliations:** 1 New York Medical College, Valhalla, New York, US; 2 Houston Methodist Hospital, Houston, Texas, US; 3 Westchester Medical Center, Valhalla, New York, US

**Keywords:** aortic stenosis, chordal rupture, mitral regurgitation, MitraClip, paravalvular leak, transcatheter aortic valve replacement

## Abstract

Transcatheter aortic valve replacement (TAVR) has become an established alternative to surgical aortic valve replacement for high-, intermediate-, and low-risk patients. Paravalvular leak (PVL) is a complication of TAVR that can be effectively treated with balloon dilation and vascular plugs. We report a case of an 86-year-old male presenting with symptomatic severe aortic stenosis. After the index TAVR procedure, mild-to-moderate PVL was noted. Two years post-operation, the patient presented with symptomatic severe PVL, which was initially treated by balloon dilation with additional volume. During balloon dilation, the balloon slipped into the left ventricle and tore a chord, leading to new severe mitral regurgitation (MR) while the PVL remained unchanged. Subsequently, an Amplatzer vascular plug II (Abbott) was successfully used to reduce the PVL to mild, and a MitraClip NTR (Abbott) was used to successfully reduce the MR to trivial. Although balloon dilation can be an effective method for reducing PVL, mitral valve chordal rupture is a rare complication if the wire is entrapped in the chordae and the balloon slips into the ventricle.

## Introduction

Transcatheter aortic valve replacement (TAVR) has become an established alternative to surgical aortic valve replacement for high-, intermediate-, and low-risk patients.^1^ One of the vulnerabilities of TAVR compared to surgical aortic valve replacement (SAVR) has been paravalvular leak (PVL). Although the incidence of PVL has declined due to improved valve designs, it remains a complication of TAVR with a reported prevalence ranging from 7% to 40%.^2^ One approach used to reduce PVL is balloon dilation. Herein, we report the pitfalls and caution that must be taken when using balloon dilation for the treatment of PVL.

## Case Report

An 86-year-old male presented to our hospital for evaluation of angina and worsening dyspnea on exertion that led to marked limitation of physical activity. These symptoms, along with the patient’s history of coronary artery disease, were consistent with a diagnosis of heart failure. The patient’s medical history included prior percutaneous coronary intervention on two occasions, mild chronic obstructive pulmonary disease, and chronic kidney disease.

Diagnostic catheterization showed 75% and 60% stenosis of the first diagonal branch of the left anterior descending artery and the middle right coronary artery, respectively. Transthoracic echocardiography showed an aortic valve area of 0.8 cm^2^, a mean gradient of 35 mm Hg, and a max velocity of 3.72, thereby meeting the criteria to be considered symptomatic severe aortic stenosis. He also had moderate aortic insufficiency (2+) and moderate mitral regurgitation (2+).

**Figure 1:**
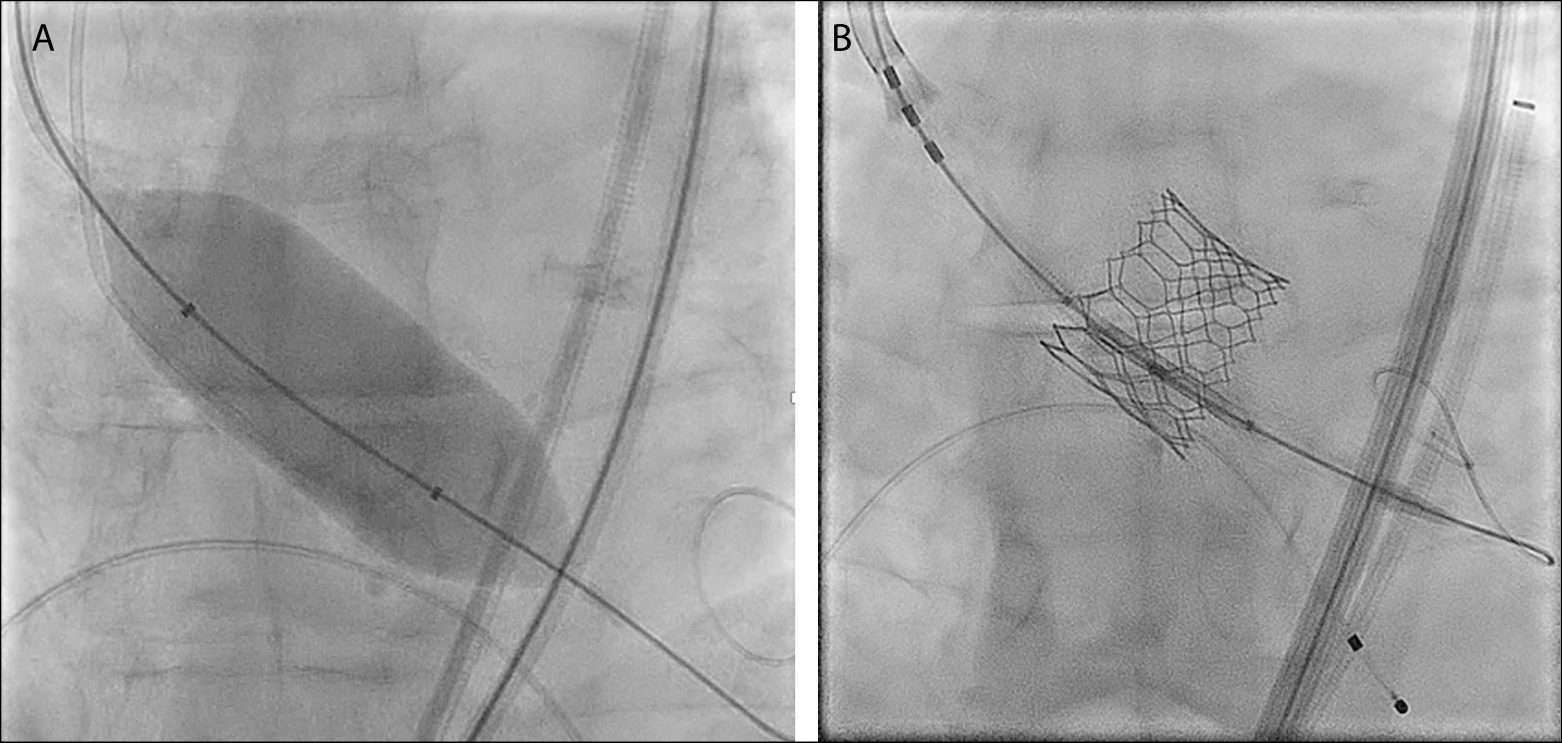
Perioperative fluoroscopy showing **(A)** dilation and **(B)** fully expanded valve.

The patient was referred for transfemoral TAVR. The Society of Thoracic Surgeons mortality risk score was 4.57%, indicating intermediate risk for TAVR. Pre-procedure anatomy characterization using multidetector computed tomography (CT) noted a large annulus with an area of 709.7 mm^2^, annular calcification, and severe valvular calcification. Due to the large aortic annulus size, the decision was made to use the largest (29 mm) Sapien 3 prosthetic valve (Edwards Lifesciences) and to deliver the catheter via right transfemoral access under conscious sedation. The valve prosthesis was undersized 8.6% to the annulus and 5.4% to the left ventricular outflow tract. Predilation, which was our routine practice at the time, was performed with 25 mm balloon aortic valvuloplasty (Figure 1A). The 29 mm S3 valve was overexpanded an additional 3 mL during deployment to account for the large annulus (Figure 1B). Postprocedural transthoracic echocardiography (TTE) showed mild to moderate PVL.

**Figure 2:**
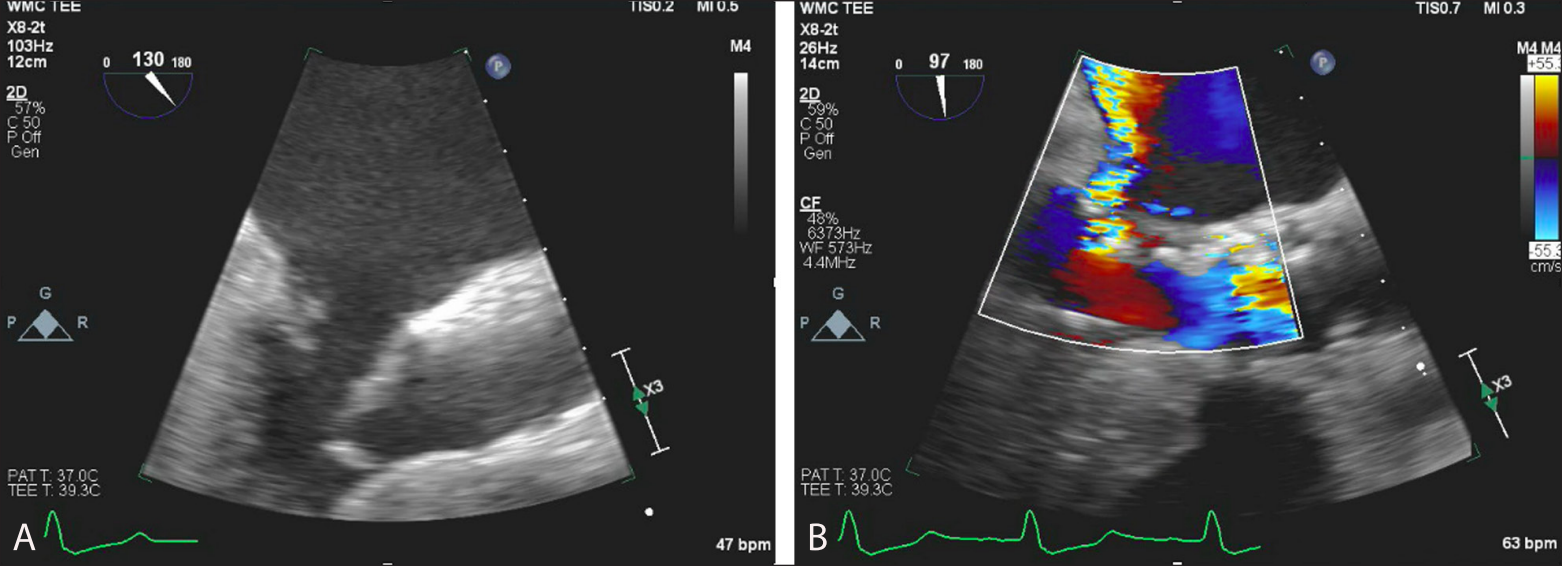
Transesophageal echocardiography showing **(A)** chordal rupture and **(B)** severe mitral regurgitation.

Two years later, the patient presented with symptoms of New York Heart Association (NYHA) class III heart failure. TTE showed severe PVL. A review of the patient’s pre-TAVR CT scan showed a medium-sized area of annular calcification with inadequate sealing—the likely location of the PVL. This was confirmed with transesophageal echocardiography (TEE). We subsequently decided to perform additional balloon dilation of the valve as an initial step to reduce the PVL followed by PVL closure with a vascular plug, if needed. With the patient under general anesthesia, we used TEE guidance to fill an Edwards balloon an additional 5 mL over nominal volume and dilated the valve, which did not improve the PVL (Video 1). The balloon had slipped into the ventricle during balloon dilation, and TEE immediately showed severe mitral regurgitation with hypotension, which was medically treated. Wire entrapment in the chordae was not noted before balloon dilation but was likely unrecognized (Figure 2).

**Video 1:** Perioperative fluoroscopy showing dilated balloon slipping into the left ventricle; also at https://youtube.com/shorts/Yj-x1ei9OCw.

**Figure 3:**
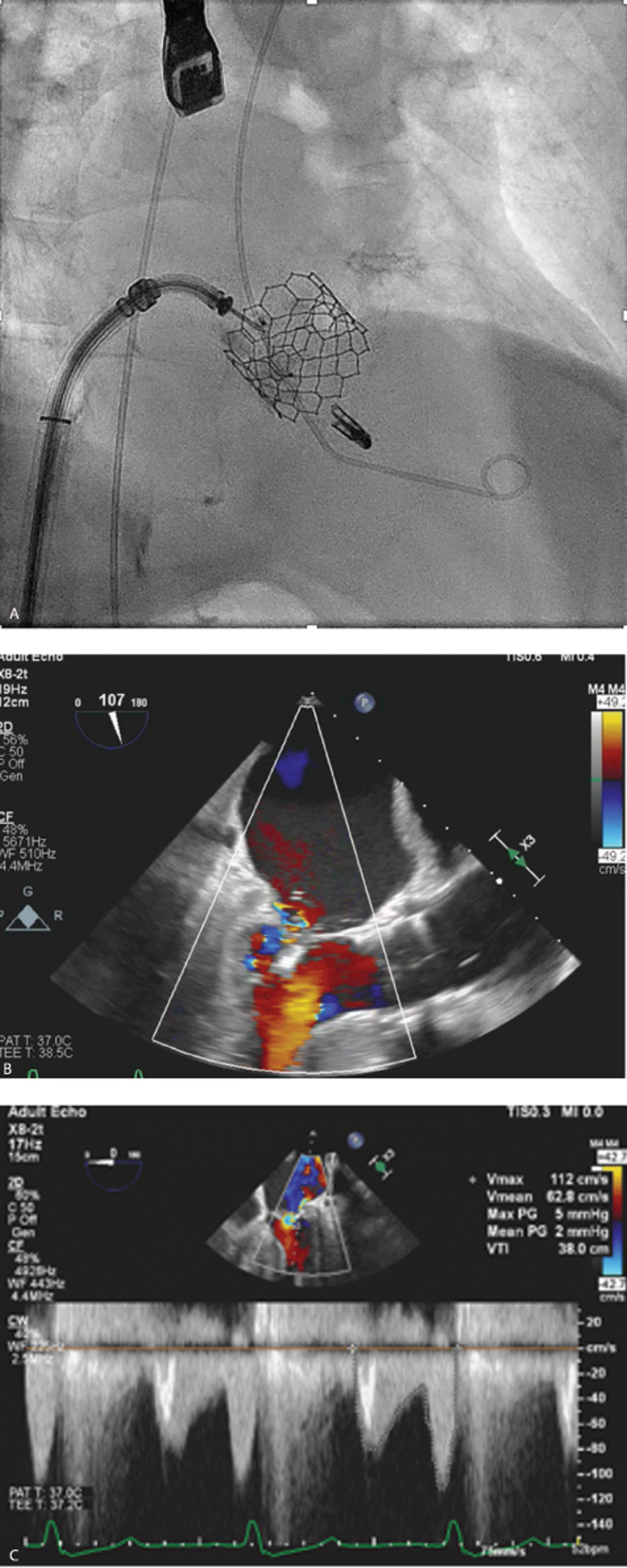
**(A)** Deployed MitraClip and **(B, C)** improved mitral regurgitation on transesophageal echocardiographic views.

The patient remained hemodynamically stable, and the PVL was successfully reduced to mild with an Amplatzer vascular plug II (Abbott). The severe mitral regurgitation was subsequently reduced to trivial with a single MitraClip NTR (Abbott) during the same procedure (Figure 3). The patient was discharged home in 48 hours. On 30-day follow-up, the patient’s heart failure improved from NYHA III to NYHA I, and he was off home oxygen and experiencing no symptoms.

## Discussion

Paravalvular leak is a known complication of TAVR, more so than after SAVR at both 1 and 2 years post procedure, and the presence of moderate or greater PVL after TAVR is associated with increased mortality.^3^ The common predictors of PVL post-TAVR are annular calcification, annular eccentricity, and undersizing or malpositioning of the device.^4^ Balloon dilation has been reported as an effective treatment for PVL arising from valvular calcification and device undersizing. Both a higher degree of valve calcification and transfemoral approach predict the need for balloon dilation, which has been shown to reduce PVL; periprocedural balloon dilation was successful in our case.^5^ In patients with extremely large annuli (> 683 mm^2^), it has been shown that despite valve undersizing, it is possible to achieve the same proportion of PVL as in smaller annuli.^6^ When dilating the valve, one must be careful to avoid wire entrapment in the mitral valve chordal apparatus, balloon slippage into the left ventricle, and damage to the mitral chordae. The mitral subvalvular complex consists of the fibrocollagenous tendinous cords, which originate from the papillary muscles.^7^ The papillary muscles are connected to the free wall of the left ventricle, while the tendinous cords attach to the free edges and ventricular aspects of the mitral leaflets. The cords are thinnest at their sites of insertion on the leaflets, thereby predisposing them to chordal rupture at that site. Due to the proximity of the aortic valve to the mitral valve apparatus, one must be cautious not to impact the mitral apparatus when intervening on the aortic valve. Although the balloon was deployed rapidly to ensure an appropriate reduction in cardiac output to facilitate safe deployment, the balloon slipped. We believe that the wire was trapped in the chordae, and the balloon slippage led to the pulling and subsequent rupturing of the chordae. We report chordal rupture related to balloon slippage during aortic valve balloon dilation as a possible rare complication that may be successfully treated with an emergency MitraClip procedure.

## Conclusion

This case highlights the importance of recognizing the various treatment options for PVL as well as their indications and pitfalls. Although balloon dilation has been shown to be an effective option to reduce PVL, the operator must use extreme caution deploying the balloon so as not to impact the mitral subvalvular structures. When considering the use of dilatation, mitral valve chordal rupture should be included as a possible periprocedural complication.
